# Deproteinized calf blood extract With or without punctal plugs versus preservative-free sodium hyaluronate for glaucoma-associated ocular surface disease: a three-arm randomized controlled trial

**DOI:** 10.3389/fmed.2026.1881940

**Published:** 2026-09-25

**Authors:** Xiao Ma, XiaoXia Li

**Affiliations:** Department of Ophthalmology, Beijing Shijitan Hospital, Capital Medical University, Beijing, China

**Keywords:** anti-glaucoma drugs, deproteinized calf blood extract, glaucoma, ocular surface disease, punctal plugs, sodium hyaluronate

## Abstract

**Introduction:**

To evaluate the short-term clinical outcomes of deproteinized calf blood extract (DCBE) with or without punctal plugs compared with preservative-free sodium hyaluronate in patients with ocular surface disease (OSD) associated with long-term anti-glaucoma therapy.

**Methods:**

Sixty patients were randomly assigned to one of three groups: Group I (DCBE plus punctal plugs), Group II (DCBE alone), and a control group (preservative-free 0.1% sodium hyaluronate). All patients continued their anti-glaucoma regimens throughout the study. Ocular symptoms, tear film parameters, and intraocular pressure (IOP) were assessed at baseline, one month, and two months post-treatment.

**Results:**

Numerical improvements in OSDI, TBUT, and CFS were observed in the DCBE-treated groups during follow-up. At 2 months, the difference between Group I and the control group favored Group I for OSDI (mean difference, −10.82; 95% CI, −20.80 to −0.84) and CFS (mean difference, −0.70; 95% CI, −1.33 to −0.07), but neither remained statistically significant after correction for multiple comparisons (Holm-adjusted *P* = .104 and.091, respectively). No significant between-group difference in TBUT was observed. Schirmer I test and meibum scores also did not differ significantly between Group I and the control group. At 2 months, IOP was lower in Group I than in the control group (mean difference, −1.90 mmHg; 95% CI, −3.10 to −0.70; Holm-adjusted *P* = .009); this was considered an exploratory finding.

**Conclusion:**

DCBE-based treatment was associated with favorable short-term changes in several ocular surface outcomes in glaucoma-associated OSD. Addition of punctal plugs was associated with favorable numerical changes, although significant between-group ocular surface differences were not demonstrated after correction for multiple comparisons. The observed IOP reduction was exploratory and should not be interpreted as evidence that OSD treatment directly improves IOP control.

## Introduction

Ocular surface disease (OSD) is a common comorbidity among patients with glaucoma receiving long-term topical therapy, with reported prevalence estimates ranging from approximately 48% to 78% (1, 2). The development and progression of glaucoma-associated OSD are multifactorial and may reflect the effects of chronic exposure to topical active agents, preservatives, frequent drop instillation, and the underlying susceptibility of the ocular surface. Benzalkonium chloride (BAK), a widely used preservative in ophthalmic preparations, has been particularly associated with epithelial toxicity and ocular surface inflammation following chronic exposure (2, 3). Consequently, patients requiring multiple topical anti-glaucoma medications may experience a substantial cumulative ocular surface burden.

The clinical consequences of OSD in patients with glaucoma extend beyond ocular discomfort. Ocular surface abnormalities may adversely affect quality of life and treatment adherence, interfere with visual field examination, and potentially compromise the outcomes of glaucoma filtration surgery. Associations between ocular surface status and intraocular pressure (IOP) control have also been reported, although the mechanisms underlying these observations remain uncertain and may involve medication adherence, tolerability, preservative exposure, or other treatment-related factors (4–8). Recognition and management of glaucoma-associated OSD have therefore become increasingly important components of long-term glaucoma care (9–12).

Managing OSD in this population can be challenging because topical IOP-lowering therapy generally cannot be discontinued. Furthermore, many patients require escalating or multidrug treatment regimens as glaucoma progresses, potentially increasing chronic ocular surface exposure to active agents and preservatives (11, 13, 14). Conventional lubricants and preservative-free tear substitutes are commonly used as first-line supportive therapies, but some patients with moderate or severe disease continue to experience persistent symptoms and ocular surface abnormalities despite these measures (9–12). Additional therapeutic approaches that can be used without interrupting essential anti-glaucoma treatment are therefore of clinical interest.

Deproteinized calf blood extract (DCBE) is a protein-free hemodialysate containing low-molecular-weight components derived from calf blood. Previous clinical studies have reported improvements in tear secretion, tear film stability, ocular pain (15), and other dry-eye-related outcomes following treatment with DCBE (16). Experimental studies have also suggested that DCBE may promote corneal epithelial wound healing and influence mucin expression in human corneal epithelial cells (17). Clinical applications of DCBE have been investigated in several ocular surface conditions, including postoperative epithelial injury, recurrent corneal erosion, graft-versus-host disease-associated dry eye, corneal epithelial defects, and other forms of dry eye disease (18–23). However, the clinical effects of DCBE in patients with OSD specifically associated with chronic anti-glaucoma therapy have not been well characterized.

Punctal occlusion is another established treatment option for dry eye disease, particularly in patients with persistent aqueous deficiency or inadequate response to lubricants (24, 25). By reducing tear drainage, punctal plugs increase tear retention and may prolong ocular surface contact with both endogenous tears and topically administered agents. However, punctal occlusion may theoretically also prolong exposure to inflammatory tear components or topical medications, making its role in glaucoma-associated OSD particularly relevant. Previous studies have reported beneficial ocular surface effects of punctal plugs in selected patients receiving topical glaucoma therapy (26, 27). Nevertheless, evidence remains limited, particularly among patients receiving multiple anti-glaucoma medications, and the potential benefit of adding punctal occlusion to an active ocular surface therapy has not been fully established.

Accordingly, the present study evaluated the short-term clinical outcomes of DCBE combined with punctal plugs, DCBE alone, and preservative-free 0.1% sodium hyaluronate in patients with glaucoma-associated OSD who continued their existing topical anti-glaucoma regimens. We assessed both subjective symptoms and objective ocular surface parameters over a 2-month treatment period. IOP was also monitored as an exploratory clinical outcome. The study was designed to determine whether DCBE-based therapy was associated with improvement in glaucoma-associated OSD and whether the addition of punctal occlusion was associated with further improvement in selected ocular surface outcomes.

## Patients and methods

### Study design and participants

This prospective, single-center, three-arm, parallel-group, randomized controlled trial was conducted at the Department of Ophthalmology, Beijing Shijitan Hospital, Capital Medical University, Beijing, China. The study protocol was approved by the Ethics Committee of Beijing Shijitan Hospital, Capital Medical University (approval No. 2018-q21) before participant recruitment. Written informed consent was obtained from all participants before enrollment, and the study was conducted in accordance with the principles of the Declaration of Helsinki. The trial was not prospectively registered in a public clinical-trials registry.

Patients presenting to the glaucoma clinic under the care of Dr. X.L. between October 1, 2023, and January 31, 2024, who had continuously used topical anti-glaucoma medications for at least 1 year were prospectively screened for ocular surface disease by an OSD specialist (Dr. X.M.). Eligibility required fulfillment of the revised 2020 Asia Dry Eye Society diagnostic criteria for dry eye disease, including the presence of subjective dry-eye symptoms and a tear break-up time (TBUT) ≤ 5 s. Exclusion criteria were a history of ocular trauma or surgery, active ocular infection, systemic autoimmune disease, contact lens use, or use of anti-inflammatory medications within the preceding 3 months.

Both eyes were examined at screening, but only one prespecified study eye per participant was included in the efficacy analysis. If both eyes met the eligibility criteria, the eye with the lower TBUT was selected. If both eyes had identical TBUT values, the right eye was selected.

### Randomization, allocation, and masking

Before participant recruitment, X.L. generated the allocation sequence using a random-number table with a 1:1:1 allocation ratio. Treatment assignments were placed in sequentially numbered, opaque, sealed envelopes prepared by X.M. The next envelope was opened only after written informed consent had been obtained and eligibility had been confirmed. However, because the enrolling investigator had access to the allocation sequence, allocation concealment was not fully maintained.

Because punctal plug insertion and the study formulations were readily distinguishable, participants and treating investigators were not masked to treatment allocation. Follow-up outcome assessments were performed by X.L., who was unaware of treatment allocation.

### Interventions

Participants were randomly allocated to three groups, with 20 participants in each group. Group I received 20% deproteinized calf blood extract (DCBE) eye gel (Xingqi Pharmaceutical Co., Ltd., Shenyang, China) four times daily together with VisiPlug™ absorbable intracanalicular punctal plugs (0.4 or 0.5 mm in diameter; Lacrimedics, Inc., WA, USA). The plugs were inserted into the superior and inferior puncta of the selected study eye. Group II received 20% DCBE eye gel alone four times daily. The control group received preservative-free 0.1% sodium hyaluronate eye drops four times daily.

All participants continued their pre-existing anti-glaucoma medications throughout the study period without planned modification. Other artificial tears and ocular lubricants were discontinued during the study. For each anti-glaucoma medication, the generic name, drug class, daily dosing frequency, preservative status, and, where ascertainable, exposure to benzalkonium chloride (BAK) were recorded.

No single primary outcome was prospectively designated; therefore, the efficacy analyses were considered exploratory. Clinical ocular surface outcomes included the Ocular Surface Disease Index (OSDI), TBUT, corneal fluorescein staining (CFS), Schirmer I test, lid-margin abnormality score, and meibum score. Intraocular pressure (IOP) was evaluated as an exploratory clinical outcome. No formal *a priori* sample-size calculation was performed. The study enrolled 60 participants, with 20 allocated to each treatment group.

### Outcome assessments

All participants completed the OSDI questionnaire and underwent a standardized ocular surface examination at baseline and at 1 and 2 months after initiation of treatment. TBUT was measured after instillation of one drop of 0.5% fluorescein. The corneal surface was observed under cobalt blue illumination, and TBUT was defined as the interval between the last complete blink and the first appearance of a dark spot in the fluorescein-stained tear film.

CFS was graded using a 0–6 scale (28). A score of 0 indicated no punctate epithelial erosions (PEE), 1 indicated 1–5 PEE, 2 indicated 6–30 PEE, and 3 indicated >30 PEE. One additional point was assigned for each of the following findings: PEE within the central 4-mm corneal zone, filamentary lesions, or confluent staining.

The Schirmer I test was performed without topical anesthesia. A Schirmer strip was placed in the conjunctival sac at the junction of the middle and lateral thirds of the lower eyelid while avoiding corneal contact. Participants closed their eyes, and the length of strip wetting was recorded after 5 min.

Lid-margin abnormality was assessed using a cumulative score ranging from 0 to 4 based on the presence of four features: irregular lid margin, lid-margin hyperemia, plugged meibomian gland orifices, and anterior or posterior displacement of the mucocutaneous junction. Each abnormality contributed one point.

Meibum expression was assessed from eight glands in the central third of the lower eyelid. Each gland was graded from 0 to 3 according to the appearance of the secretion and the pressure required for expression, giving a total score ranging from 0 to 24, with higher scores indicating greater meibomian gland dysfunction.

IOP was measured using Goldmann applanation tonometry. To minimize the potential influence of diurnal variation, IOP measurements for each participant were scheduled at approximately the same time of day at baseline and at each follow-up visit.

All 60 randomized participants completed the 2-month follow-up and were analyzed according to their originally assigned treatment groups. No outcome measurements were missing at the 1- or 2-month follow-up visits.

### Statistical analysis

Statistical analyses were performed using SPSS version 19.0 (IBM Corp., Armonk, NY, USA). Continuous variables are presented as mean ± standard deviation (SD), and categorical variables as number and percentage, as appropriate. Baseline demographic, ocular-surface, and anti-glaucoma treatment characteristics were summarized descriptively according to randomized treatment group. Statistical significance testing of baseline characteristics was not performed to assess the success of randomization.

All randomized participants were analyzed according to their originally assigned treatment groups. Because all 60 participants completed the 1- and 2-month follow-up visits and no outcome data were missing, no imputation for missing values was required.

Longitudinal treatment effects were evaluated using mixed-effects models to account for repeated measurements within participants. For each outcome, the post-baseline measurements at 1 and 2 months were included as the dependent variable. Treatment group, follow-up visit, and the treatment-group-by-visit interaction were included as fixed effects, and the corresponding baseline value of the outcome was included as a covariate. Participant was included as a random effect to account for within-participant correlation. This baseline-adjusted approach was used for all longitudinal treatment comparisons and accounted for observed baseline differences, including the lower baseline TBUT in Group I.

From each model, adjusted between-group treatment effects were estimated separately at the 1- and 2-month follow-up visits and are reported as adjusted mean differences with 95% confidence intervals (CIs). Pairwise comparisons included Group I versus the control group, Group II versus the control group, and Group I versus Group II. For each outcome at each follow-up visit, these three pairwise comparisons were considered one multiplicity family, and the corresponding *P*-values were adjusted using the Holm procedure.

Model assumptions were assessed by examination of residual distributions and model diagnostics. Where the assumptions of a linear mixed-effects model were not adequately satisfied, an appropriate generalized mixed-effects model or transformation was considered according to the distribution and scale of the outcome. Categorical variables were summarized descriptively and, where inferential comparison was appropriate, were analyzed using the chi-square test or Fisher's exact test.

All statistical tests were two-sided, and a Holm-adjusted *P* < 0.05 was considered statistically significant for pairwise treatment comparisons. Because no single primary efficacy outcome was prospectively designated and no formal *a priori* sample-size calculation was performed, efficacy analyses were considered exploratory; therefore, effect estimates and 95% CIs were considered together with *P*-values when interpreting the findings.

## Results

Baseline demographic and clinical characteristics are summarized in Table 1. The three groups were generally similar with respect to age, sex, OSD severity, and number of anti-glaucoma medications. Group I had a lower baseline TBUT than the control group (2.15 ± 1.10 vs. 3.08 ± 1.29 s). All 60 randomized participants completed the 2-month follow-up and were analyzed according to their originally assigned treatment groups (Figure 1). Anti-glaucoma medication exposure was broadly similar across the three groups, including the proportions receiving prostaglandin analogues, *β*-blockers, carbonic anhydrase inhibitors, *α*2-adrenergic agonists, fixed-combination therapy, preservative-free medications, and BAK-containing medications, as well as the mean number of daily topical instillations (Table 1).

**Table 1:** Demographic characteristics of cases and control groups.

Characteristic	Group I: DCBE + punctal plugs (n=20)	Group II: DCBE (*n*=20)	Control: preservative-free 0.1% sodium hyaluronate (*n*=20)
Age, years	60.05 ± 10.81	59.50 ± 9.94	57.40 ± 12.36
Sex, male/female	8/12	6/14	8/12
OSD severity, n (%)			
Mild	3 (15)	5 (25)	5 (25)
Moderate	4 (20)	5 (25)	4 (20)
Severe	13 (65)	10 (50)	11 (55)
Number of antis-glaucoma medications, n (%)			
1	7 (35)	10 (50)	6 (30)
2	8 (40)	6 (30)	8 (40)
≥3	5 (25)	4 (20)	6 (30)
Anti-glaucoma medication characteristics			
Prostaglandin analogue, n (%)	13 (65)	13 (65)	14 (70)
*β*-blocker, n (%)	11 (55)	10 (50)	11 (55)
Carbonic anhydrase inhibitor, n (%)	7 (35)	6 (30)	8 (40)
*α*2-adrenergic agonist, n (%)	5 (25)	4 (20)	5 (25)
Fixed-combination therapy, n (%)	7 (35)	6 (30)	7 (35)
Receiving ≥1 preservative-free medication, n (%)	3 (15)	4 (20)	3 (15)
Receiving ≥1 BAK-containing medication, n (%)	16 (80)	15 (75)	16 (80)
Daily topical instillations per study eye, mean ± SD	2.8 ± 1.3	2.6 ± 1.2	2.9 ± 1.4

**Figure 1:**
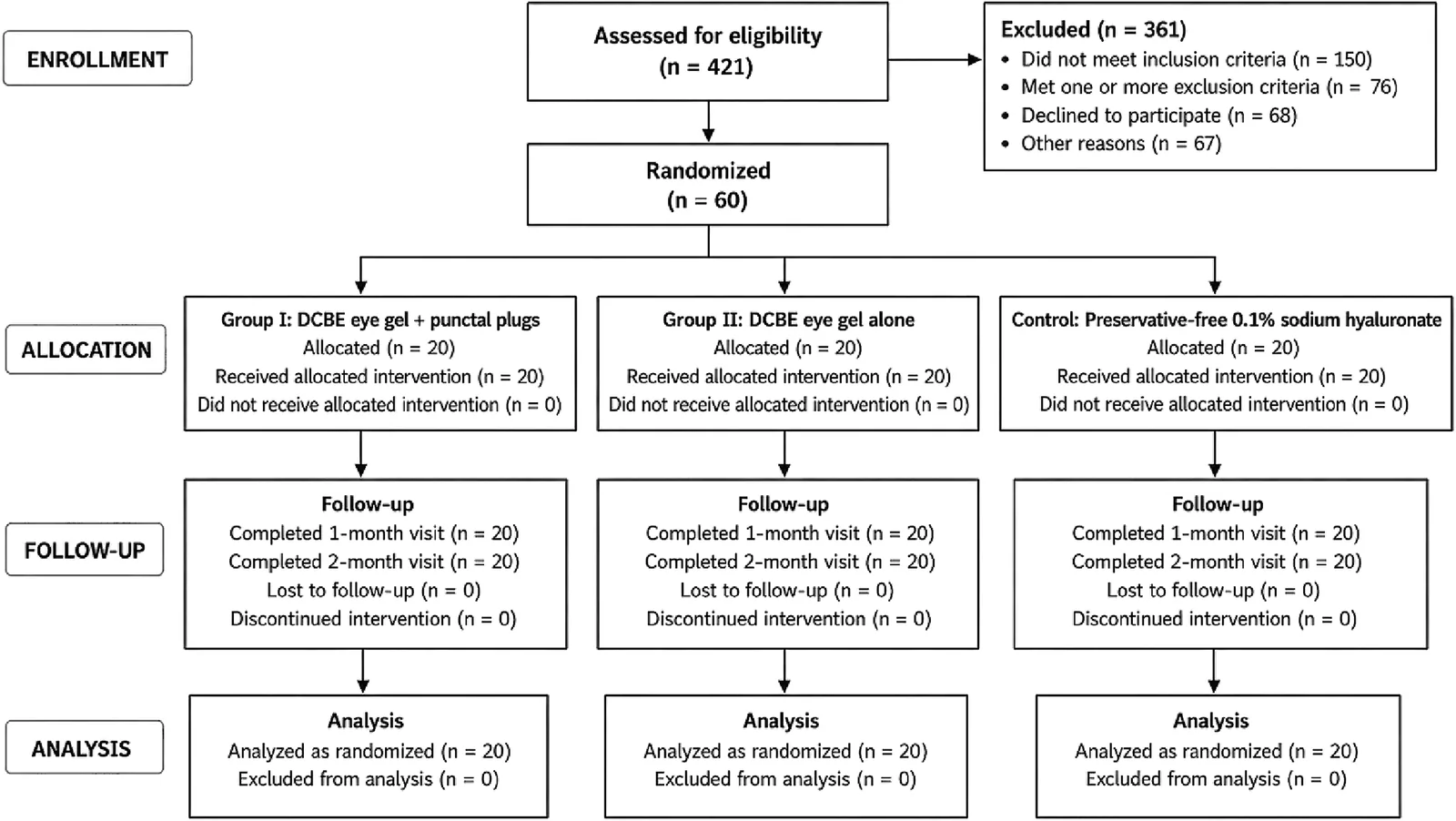
CONSORT flow diagram of participant enrollment, allocation, follow-up, and analysis.

OSDI scores decreased during follow-up in all three groups, with greater numerical reductions in the two DCBE-treated groups (Figure 2). In Group I, mean OSDI decreased from 38.43 ± 16.13 at baseline to 27.51 ± 12.64 at 1 month and 19.99 ± 10.14 at 2 months. Corresponding values in Group II were 33.06 ± 18.57, 24.24 ± 12.85, and 21.14 ± 10.26, whereas those in the control group were 35.91 ± 20.95, 29.25 ± 20.19, and 30.81 ± 19.32. At 2 months, the mean difference between Group I and the control group was −10.82 points (95% CI, −20.80 to −0.84); however, this comparison did not remain statistically significant after correction for multiple comparisons (Holm-adjusted *P* = .104).

**Figure 2:**
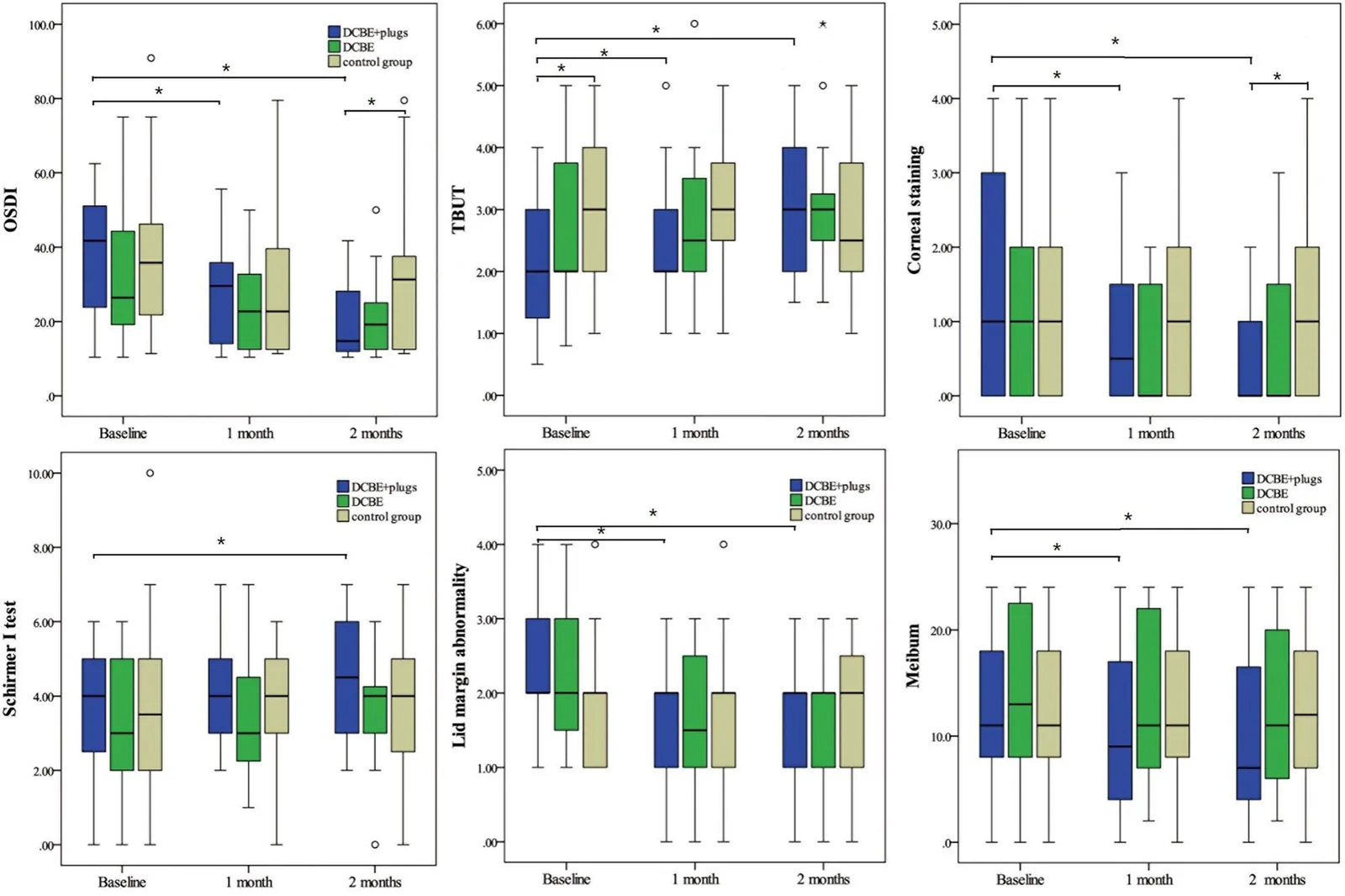
OSDI and ocular surface parameters at baseline and at 1 and 2 months after treatment. Values are shown for Group I (DCBE plus punctal plugs), Group II (DCBE alone), and the control group (preservative-free 0.1% sodium hyaluronate). Numerical changes in OSDI, TBUT, CFS, Schirmer I test, lid-margin abnormality, and meibum score are shown over the 2-month follow-up. Between-group statistical comparisons are reported in Table 2. OSDI, Ocular Surface Disease Index; TBUT, tear break-up time; CFS, corneal fluorescein staining; DCBE, deproteinized calf blood extract.

**Table 2:** Clinical parameters before and after treatment in OSD.

Outcome/visit	Group I: DCBE + punctal plugs (*n*=20)	Group II: DCBE (*n*=20)	Control (*n*=20)	Adjusted mean difference (95% CI)	Holm-adjusted P
OSDI					
Baseline	38.43 ± 16.13	33.06 ± 18.57	35.91 ± 20.95	—	—
1 month	27.51 ± 12.64	24.24 ± 12.85	29.25 ± 20.19	—	—
2 months	19.99 ± 10.14	21.14 ± 10.26	30.81 ± 19.32	−10.82 (−20.80 to −0.84)	.104
TBUT, s					
Baseline	2.15 ± 1.10	2.54 ± 1.27	3.08 ± 1.29	—	—
1 month	2.50 ± 1.11	2.85 ± 1.11	3.05 ± 1.17	−0.55 (−1.28 to 0.18)	.407
2 months	3.13 ± 1.12	3.08 ± 1.03	2.82 ± 1.10	0.31 (−0.40 to 1.02)	1.000
CFS score					
Baseline	1.50 ± 1.50	1.35 ± 1.27	1.30 ± 1.34	—	—
1 month	0.80 ± 0.95	0.70 ± 0.87	1.15 ± 1.23	—	—
2 months	0.45 ± 0.61	0.75 ± 1.07	1.15 ± 1.23	−0.70 (−1.33 to −0.07)	.091
Schirmer I test, mm					
Baseline	3.55 ± 1.79	3.30 ± 1.78	3.68 ± 2.41	—	—
1 month	4.08 ± 1.47	3.45 ± 1.71	3.70 ± 1.59	—	—
2 months	4.53 ± 1.68	3.63 ± 1.40	3.80 ± 1.93	0.73 (−0.43 to 1.89)	.420
Lid-margin abnormality score					
Baseline	2.30 ± 0.80	2.15 ± 0.93	1.85 ± 0.75	—	—
1 month	1.80 ± 0.89	1.55 ± 1.09	1.75 ± 0.79	—	—
2 months	1.75 ± 0.79	1.50 ± 1.00	1.80 ± 1.01	—	—
Meibum score					
Baseline	12.40 ± 7.29	14.20 ± 7.43	12.40 ± 7.29	—	—
1 month	10.55 ± 7.64	13.20 ± 7.47	11.90 ± 7.36	—	—
2 months	9.75 ± 7.35	13.00 ± 7.46	12.30 ± 7.26	−2.55 (−7.23 to 2.13)	.553
IOP, mmHg					
Baseline	16.47 ± 3.02	15.94 ± 1.89	15.95 ± 2.19	—	—
1 month	15.05 ± 1.64	14.59 ± 1.65	15.37 ± 1.95	—	—
2 months	13.99 ± 1.37	14.78 ± 1.75	15.89 ± 2.24	−1.90 (−3.10 to −0.70)	.009

TBUT increased during follow-up in both DCBE-treated groups. Group I increased from 2.15 ± 1.10 s at baseline to 2.50 ± 1.11 s at 1 month and 3.13 ± 1.12 s at 2 months, whereas Group II increased from 2.54 ± 1.27 to 2.85 ± 1.11 and 3.08 ± 1.03 s, respectively. The control group showed little change, with values of 3.08 ± 1.29, 3.05 ± 1.17, and 2.82 ± 1.10 s. The difference between Group I and the control group was −0.55 s at 1 month (95% CI, −1.28 to 0.18; Holm-adjusted *P* = .407) and 0.31 s at 2 months (95% CI, −0.40 to 1.02; Holm-adjusted *P* = 1.000), indicating no significant between-group difference.

CFS scores decreased numerically in both DCBE-treated groups. In Group I, mean CFS decreased from 1.50 ± 1.50 at baseline to 0.80 ± 0.95 at 1 month and 0.45 ± 0.61 at 2 months. At 2 months, the difference between Group I and the control group was −0.70 (95% CI, −1.33 to −0.07). Although the numerical difference favored Group I, it was not statistically significant after correction for multiple comparisons (Holm-adjusted *P* = .091).

Schirmer I test values increased numerically in Group I from 3.55 ± 1.79 mm at baseline to 4.53 ± 1.68 mm at 2 months. The 2-month difference between Group I and the control group was 0.73 mm (95% CI, −0.43 to 1.89; Holm-adjusted *P* = .420). Similarly, the meibum score decreased from 12.40 ± 7.29 at baseline to 9.75 ± 7.35 at 2 months in Group I, but the difference compared with the control group was not statistically significant (mean difference, −2.55; 95% CI, −7.23 to 2.13; Holm-adjusted *P* = .553). Thus, neither Schirmer I test nor meibum score demonstrated a significant between-group difference.

Mean IOP decreased from 16.47 ± 3.02 mmHg at baseline to 15.05 ± 1.64 mmHg at 1 month and 13.99 ± 1.37 mmHg at 2 months in Group I. In Group II, IOP decreased from 15.94 ± 1.89 to 14.59 ± 1.65 and 14.78 ± 1.75 mmHg, respectively, whereas the control group showed little overall change. At 2 months, Group I had a lower mean IOP than the control group, with a between-group difference of −1.90 mmHg (95% CI, −3.10 to −0.70), which remained statistically significant after correction for multiple comparisons (Holm-adjusted *P* = .009). Because IOP was an exploratory outcome, this finding should be interpreted as an association rather than evidence that treatment of OSD directly improved IOP control.

Regarding punctal plug retention and safety, two plugs (5%) were lost and three (7.5%) were partially extruded during follow-up. Partially extruded plugs were repositioned into the vertical segment of the canaliculus using a flushing needle, and lost plugs were replaced at the follow-up visit. One patient (5%) experienced transient epiphora, which resolved spontaneously within 1 week. No participant reported persistent ocular discomfort or requested plug removal.

## Discussion

In this randomized three-arm study, DCBE-based treatment was associated with short-term improvement in several symptoms and signs of glaucoma-associated OSD while patients continued their topical anti-glaucoma therapy. Numerical improvements in OSDI, TBUT, and CFS were observed in the DCBE-treated groups, particularly in the group receiving DCBE combined with punctal plugs. However, after correction for multiple comparisons, the between-group differences in OSDI, TBUT, and CFS between Group I and the control group were not statistically significant. These findings therefore suggest a potential ocular surface benefit but should be interpreted as exploratory rather than confirmatory. The sodium hyaluronate group showed symptomatic improvement but relatively limited changes in objective ocular surface signs. The small sample size and multiplicity-adjusted analyses should be considered when interpreting the magnitude and statistical certainty of these effects.

Glaucoma-associated OSD has a multifactorial pathophysiology involving reduced corneal sensitivity, abnormalities of the subbasal nerve plexus, epithelial injury, conjunctival goblet cell loss, and meibomian gland inflammation. Together, these abnormalities may contribute to aqueous tear deficiency, mucin disturbance, blepharitis, and meibomian gland dysfunction (9, 28). Previous studies have demonstrated that ocular surface symptoms and signs may persist in patients receiving chronic anti-glaucoma therapy despite treatment with conventional lubricants (12, 29). In the present study, the preservative-free 0.1% sodium hyaluronate group showed improvement in OSDI scores but relatively little change in objective ocular surface parameters during the 2-month follow-up. Most participants (47/60, 78.3%) had moderate-to-severe OSD at baseline, with an overall mean OSDI score of approximately 35.8, consistent with the substantial ocular surface burden previously reported among patients receiving long-term topical glaucoma therapy (30). Blood-derived ocular surface treatments have been used for moderate-to-severe OSD (31), and DCBE has been investigated in several ocular surface conditions (18–23). To our knowledge, the present study is among the first to specifically evaluate DCBE in glaucoma-associated OSD.

The mean OSDI score decreased from 38.43 to 19.99 in the DCBE-plus-punctal-plug group and from 33.06 to 21.14 in the DCBE-alone group. Although the 2-month difference between Group I and the control group favored Group I, it did not remain statistically significant after correction for multiple comparisons. Thus, the reduction in OSDI should be interpreted as a favorable numerical trend rather than definitive evidence of superiority. Previous studies have demonstrated alterations in corneal subbasal nerves among patients receiving chronic anti-glaucoma medications, including reduced nerve density and length and increased tortuosity (30, 32), and such neural abnormalities have been associated with ocular surface symptoms (33). Deng et al. reported that DCBE promotes the regeneration of corneal subbasal nerves following laser *in situ* keratomileusis (22), and Qu et al. identified neuroprotective effects of DCBE in Alzheimer's disease models (34). DCBE contains over 200 bioactive components and is clinically approved for the treatment of various nerve injury-related disorders. These findings provide a possible biological explanation for the symptomatic improvement observed with DCBE; however, corneal nerve morphology or function was not measured in the present study, and a nerve-repair mechanism therefore cannot be established from our data.

As a chronic, sight-threatening condition requiring lifelong management, glaucoma depends heavily on patient compliance and adherence. Effective symptom relief is critical to maintaining treatment commitment, particularly among patients undergoing complex multi-drug regimens (8, 9, 28). Although improved ocular comfort could theoretically support treatment adherence, adherence was not formally measured in the present study and this relationship cannot be inferred from our findings.

Clinical studies have demonstrated that DCBE eye drops enhance tear secretion and stabilize the tear film in patients with DED (15). In the present study, TBUT increased numerically during follow-up in both DCBE-treated groups. However, the between-group differences between Group I and the control group were not statistically significant after correction for multiple comparisons, and interpretation is further complicated by the lower baseline TBUT in Group I. Nam et al. reported that DCBE upregulates the expression of both membrane-associated and secretory mucins in primary human corneal epithelial cells (17). Mucins, which are major secretory products of conjunctival goblet cells, play essential roles in ocular surface lubrication, clearance, and barrier maintenance (35). Long-term use of anti-glaucoma drugs containing BAK induces apoptosis of these goblet cells, resulting in reduced mucin production (2, 3). DCBE-induced changes in mucin expression therefore represent a plausible mechanism that could contribute to tear-film improvement. However, mucin expression and goblet-cell density were not assessed in this study, and this mechanism remains speculative. Furthermore, compared with 0.3% sodium hyaluronate and basic fibroblast growth factor (bFGF), DCBE has shown efficacy in hydrating the ocular surface and promoting corneal epithelial repair (16, 21).

Punctal plugs contribute to the management of OSD by extending the retention time of lubricants on the eye, increasing tear volume to reduce mechanical friction (24), and supporting corneal nerve regeneration (36). Meanwhile, by blocking tear drainage, punctal plugs also potentially prolong or exacerbate ocular surface inflammation. Therefore, while some experts recommend initiating punctal plug insertion only after ocular surface inflammation has been adequately controlled, others suggest that earlier intervention may lead to more effective management of DED. Sherwin et al. and Chen et al. have reported supplementary benefits of punctal plugs in glaucoma patients on monotherapy (26, 27), but whether similar benefits can be achieved in patients using multiple anti-glaucoma drugs, it remains unclear.

In this study, 65% (13/20) of the participants in Group I were using two or more anti-glaucoma drugs. Following plug insertion, numerical improvements were observed in OSDI, TBUT, and CFS during follow-up. However, these outcomes were not statistically superior to the control group after correction for multiple comparisons. Moreover, because the study did not include a punctal-plug-only or sham-procedure group, the independent contribution of punctal occlusion cannot be distinguished from the effect of DCBE or a possible interaction between the two interventions. Accordingly, our findings suggest that adding punctal plugs to DCBE may provide additional or more sustained benefit in selected patients, but they do not establish the independent efficacy of punctal plugs.

Tong et al. observed a transient increase in tear inflammatory cytokines one week after plug insertion, which returned to baseline within three weeks (37). Changes in tear proteins following punctal occlusion have also been reported (38). These findings indicate that punctal occlusion may modify the ocular surface environment in complex ways rather than simply increasing tear volume. Given that ocular surface inflammation in glaucoma patients often persists because of continuous topical drug exposure, delaying punctal occlusion until inflammation is completely absent may not always be practical (39). Nevertheless, the present study was not designed to determine the optimal timing of plug insertion in relation to ocular surface inflammation. Therefore, early plug insertion should be considered a potentially useful strategy rather than a conclusion established by the present trial.

An exploratory reduction in IOP was observed during follow-up, and the 2-month difference between Group I and the control group remained statistically significant after correction for multiple comparisons (mean difference, −1.90 mmHg; 95% CI, −3.10 to −0.70; Holm-adjusted *P* = .009). Previous reports by Batra et al. and Dubulle et al. have described associations between treatment of ocular surface disease and changes in IOP in patients with glaucoma (6, 7). However, this result should not be interpreted as evidence that improvement of the ocular surface directly caused better IOP control. Punctal occlusion may prolong ocular surface contact with topical anti-glaucoma medications and alter their ocular absorption (26, 27). Other unmeasured factors, including medication adherence, drop-instillation technique, drug class, preservative exposure, and short-term IOP variability, may also have contributed (6, 7, 9). Although IOP measurements were scheduled at approximately the same time of day at each visit, full diurnal IOP profiles were not obtained. Notably, four patients with baseline IOP around 20 mmHg exhibited a decrease of more than 5 mmHg after treatment; however, this observation was *post hoc* and involved a very small number of participants and should therefore be considered hypothesis-generating. Larger studies specifically designed to evaluate IOP, adherence, medication exposure, and the pharmacokinetic effects of punctal occlusion are needed to confirm this finding.

For patients with severe MGD, adjunct therapies such as meibomian gland probing, short-term steroid treatment, oral azithromycin, or omega-3 supplementation have been described (6, 7, 9, 28, 40). These therapies were not evaluated as study interventions in the present trial, and no conclusions regarding their comparative or combined efficacy with DCBE or punctal occlusion can be drawn from our data.

The present study has several limitations. First, this was a single-center study with only 20 participants per treatment group, and no formal *a priori* sample-size calculation or single primary efficacy outcome was prospectively designated; therefore, the efficacy analyses should be considered exploratory. Second, the trial was not prospectively registered in a public clinical-trials registry. Third, because punctal plug insertion and the study formulations were readily distinguishable, participants and treating investigators could not be masked, which may have introduced performance bias. Although follow-up outcome assessment was performed without knowledge of treatment allocation, complete masking could not be guaranteed because of the nature of the intervention. Fourth, the absence of a punctal-plug-only or sham-procedure group prevents determination of the independent effect of punctal occlusion. Fifth, participants continued their pre-existing anti-glaucoma medications, and differences in drug class, dosing frequency, preservative status, and BAK exposure may have influenced ocular surface outcomes. Sixth, medication adherence and drop-instillation technique were not formally measured, limiting interpretation of the exploratory IOP findings. Finally, follow-up was limited to 2 months, and longer-term efficacy, safety, and punctal plug retention remain uncertain.

Despite these limitations, the findings suggest that DCBE-based treatment may provide short-term improvement in glaucoma-associated OSD while anti-glaucoma therapy is continued. Addition of punctal plugs was associated with favorable numerical changes in several ocular surface outcomes, although these differences did not remain statistically significant after correction for multiple comparisons and the independent effect of punctal occlusion could not be established. The significant exploratory reduction in IOP observed in Group I warrants further investigation but should not be interpreted as evidence of a causal effect of OSD treatment on IOP control. Larger, prospectively registered studies with adequate statistical power, longer follow-up, and detailed characterization of anti-glaucoma medication and preservative exposure are required to confirm these findings.

## Data Availability

The original contributions presented in the study are included in the article/Supplementary Material, further inquiries can be directed to the corresponding author.
